# Sustaining Behavior Changes Following a Venous Leg Ulcer Client Education Program

**DOI:** 10.3390/healthcare2030324

**Published:** 2014-09-04

**Authors:** Charne Miller, Suzanne Kapp, Lisa Donohue

**Affiliations:** 1 La Trobe University Alfred Health Clinical School, Level 4, The Alfred Centre, 99 Commercial Road, Prahran, VIC 3181, Australia; 2 RDNS Institute, 31 Alma Road, St. Kilda, VIC 3182, Australia; E-Mail: skapp@rdns.com.au; 3 Faculty of Medicine and Health Sciences, School of Nursing an Midwifery, Monash University, Peninsula Campus, McMahons Rd., Frankston, VIC 3199, Australia; E-Mail: ldonohue@bluep.com

**Keywords:** chronic disease, venous leg ulcer, wound, self-management, health behaviors, behavior change

## Abstract

Venous leg ulcers are a symptom of chronic insufficiency of the veins. This study considered the sustainability of behavior changes arising from a client focus e-Learning education program called the “Leg Ulcer Prevention Program” (LUPP) for people with a venous leg ulcer. Data from two related studies were used to enable a single sample (*n *= 49) examination of behavior maintenance across an average 8 to 9 months period. Physical activity levels increased over time. Leg elevation, calf muscle exercises, and soap substitute use were seen to fluctuate over the follow up time points. The use of a moisturizer showed gradual decline over time. The provision of a client-focused venous leg ulcer program was associated with behavior changes that had varied sustainability across the evaluation period.

## 1. Introduction

### 1.1. Venous Leg Ulcers: A Common and Costly Wound

Approximately 70% of all lower leg ulcers are venous leg wounds [1]. Venous leg ulcers affect 0.1%–1.1% of people [2], with a prevalence of up to 3% for people aged over 60 years [1]. It is a condition associated with significant morbidity and impact on quality of life [3,4,5,6,7,8,9,10,11]. It has been estimated to cost $3 billion Australian dollars annually [12].

### 1.2. A Chronic Condition, a Complex Treatment

Venous leg ulcers are a symptom of peripheral vascular disease, and more specifically of chronic venous insufficiency. Parallels can be readily drawn between chronic venous insufficiency and characteristics of other chronic diseases as this condition is typically caused by multiple factors associated with a person’s genetics, environment and lifestyle [13]. Consistent with the profile of chronic disease [13], venous leg ulcers may have complex causality, multiple risk factors and cause functional disability. These wounds can take an extended time to heal and there is a high recurrence rate. For older people who have comorbid conditions, corrective surgery to fix the underlying problem with the veins is not usually a treatment option.

The current best practice treatment of venous disease involves the use of compression therapy in conjunction with optimizing the conduct of a number of lifestyle behaviors. Standards for venous leg ulcer management and prevention [14] advocate the use of the highest level of compression therapy tolerable, regular activity, leg elevation during periods of inactivity, heel raises and squats to strengthen the calf muscle, a healthy diet, adequate “good” fluid intake, and the use of a moisturizer and soap substitute to promote skin hydration. These treatments apply not only when an individual has a venous leg ulcer but also post-healing in order to prevent the ulcer from recurring. With a recurrence rate of 69% [15], repeated episodes of care is a significant issue for those affected and health professionals engaged in the wound management field.

Concordance with the recommended treatment and healthy behaviors is for people with venous disease, alike other chronic conditions, not always well adopted or adhered to [16,17]. A number of programs encouraging effective self-management including leg clubs [18], the “Lively Legs” Program [19,20], and other e-Learning initiatives [21] have been developed for use in venous leg ulcer care and prevention to optimize the uptake of recommended strategies and these have been shown to be acceptable to clients [21,22,23] and nurses [24]. While these programs have improved knowledge, physical activity, skin care, and compression use, no program has been effective in all of the targeted domains [19,21,23]. Although important efforts have been made by client education innovators in the wound management field, self-management research and innovation in the wound management field is still in its infancy when compared to other clinical conditions. Transferring knowledge gained in other clinical areas can assist the wound management field [25]. There are however gaps in knowledge that effect all clinical areas. Two particular gaps to be considered by this paper is the need to further understand the efficacy of interventions that promote multiple behavior changes and how these behaviors changes can be maintained after the intervention is finished.

Historically, the policy and evidence base regarding self-management programs has focused on changes for a single condition [26,27,28]. What is increasingly recognized is that programs that target multiple behavior changes are necessary not only to adequately address management of the chronic condition but due to individuals having multiple morbidities [28]. It is common for people, especially later in life, to have multiple chronic health conditions. Almost half (49%) of Australians living in the community aged 65–74 years have five or more long-term conditions, a rate which increases to 70% for people aged 85 years and over [29]. Many of these chronic conditions benefit from the same healthy lifestyle behaviors such as being physically active, drinking “good” fluids, and eating a nutritionally complete diet. As such, a management plan that considers the collective sum of self-management practices can consolidate recommendations that might otherwise overwhelm. Indeed, it has been suggested that a cognitive representation of illness/es and their management enables adaptions and inclusions arising from shifting health priorities [28]. That is, an integrated but broad understanding of the disease function and how healthy behaviors facilitate the management of the disease can help the individual accommodate variation arising from life or health events that optimizes their effective management of multiple morbidities. As the salience of venous disease management can be lessened as time since healing increases and in the presence of other life threatening diseases, transferences across other morbidities may be one approach wound clinicians use to optimize self-management practices.

The effectiveness of strategies to target multiple behavior change is still being unraveled. Behavior changes in multiple domains have been described as complementary or compensatory [30]. On one hand there could be competing effects between health behaviors where gain in one domain is at the cost of losses in another area [31]. In a study of a multiple component intervention including weight management, activity, stress management, smoking and social support intervention for women this issue was examined and was not found to be the case [31]. Alternatively, by spreading the focus on multiple behaviors the potential gains are diluted [31]. Certainly, the perceived incongruence between recommendations for people with venous disease to be physical active as well as to rest and elevate their leg has been noted as undermining the conduct of one or both of these behaviors [23,32,33]. There is, on the other hand, quite a few studies to support the suggestion that behavior change in one domain can in fact enable change in another [34,35,36,37,38]. Systematic review evidence of the effectiveness of multiple behavior change interventions suggest positive outcomes can be achieved but these are rarely uniform across the behavior domains being targeted [39,40,41]. More research is required to identify the circumstances in which multiple component interventions are effective.

Another challenge for the field is the absence of research considering the maintenance of behavior changes. In 2012, the results of a randomized controlled trial assessing the effectiveness of the “Lively Legs” program [20] for with people with primarily venous leg ulcers was published which assessed the performance of physical activity, use of compression therapy and wound progress over an 18 months period [19]. Participants in the “Lively Legs” program were going on more frequent short walks, engaged in leg exercises, and had better wound healing, with no difference observed in compression use and in longer duration walking. It is both exciting and promising that some behavior change was achieved, sustained, and the research team implemented such a longer term follow up of study participants.

In 2011, a systematic review was published that considered maintenance that was achieved in 157 studies assessing the effects of a dietary and/or physical activity intervention [42]. Only 35% reported maintenance outcomes up to three months, and only 10% up to one year, although of these studies maintenance rates were promising with 72% achieving maintenance [42]. Of the 29 studies that met the eligibility criteria, the range of health conditions was considerable including healthy adults, type 2 diabetes mellitus, osteoarthritis, chronic back pain, breast cancer and coronary heart disease. The study found that the only sample characteristic that influenced maintenance of a dietary and/or physical activity intervention was gender. That is, studies targeting women revealed less effective maintenance of behavior change. No differences in the degree of maintenance achieved with programs targeting healthy adults and programs targeting people with a chronic health condition [42].

More generally, this “decay of impact” [43,44] whilst not universal, is considered to be the norm [31,44,45,46]. To advance how maintenance of behavior change is understood and how health professionals can most effectively empower people to self-manage their chronic condition/s it has been suggested that maintenance be conceptualized as a process and quite possibility a separate psychological process to behavior change [46]. Support of this latter contention emerges from a review of a physical activity program among cardiac patients that found actions plans were more influential during the early rehabilitation process, while coping plans were more instrumental as rehabilitation progressed [47]. Alike chronic disease inquiry in general, the longevity of behavior changes arising from venous leg ulcer self-management programs is an area in need of investigation.

In 2007, clinicians employed by Royal District Nursing Service (RDNS), a community nursing and health provider in Australia, provided the impetus for developing the Leg Ulcer Prevention Program (LUPP). LUPP was conceived in the belief that better outcomes for clients with leg ulcers could be facilitated by innovative, structured and standardized client education and that strategies to increase client involvement in their management were warranted. A pre- and post-evaluation of the LUPP education showed that client knowledge, skin care, physical activity and compression therapy use increased after the education, and that nurses and clients expressed high satisfaction with the program [19,21]. The aim of this investigation was to ascertain the sustainability of behavior changes arising from this client focused e-learning education program for people with a venous leg ulcer.

## 2. Methods

### 2.1. Design

To enable this analysis, data from two related studies were combined to enable a prospective single sample cohort study. The first study was the LUPP pilot study (*n *= 155) in which performance of a variety of health behaviors were evaluated before (pre) and after (post) the delivery of a six part e-learning program with clients of a community nursing service in Australia. All of these study participants had an active leg ulcer at the time of the study. Forty-nine of these participants upon healing, subsequently participated in a randomized controlled trial (RCT) (total *n *= 100) comparing wound recurrence associated with the use of a moderate and a high compression stocking. Evaluation of health behaviors amongst this sample occurred at baseline, and 13 weeks and 26 weeks after recruitment. Thus, in total there were five time points available for 49 participants in total to examine how sustainable the behavior changes associated with the LUPP education were over time.

By combining these two data sources that used the same measures at each time point, it became possible to examine the sustainability of behavior change for people who participated in both the preliminary LUPP pilot evaluation and the RCT. Though using RCT data this investigation is not an experimental study but rather a single sample longitudinal appraisal of health behavior maintenance.

### 2.2. The Leg Ulcer Prevention Program (LUPP) 

LUPP is a standardized e-Learning client education package that delivers best practice recommendations for venous leg ulcer management. LUPP was developed by a multidisciplinary team including wound management clinical nurse consultants, researchers, allied health practitioners, e-Learning experts, marketing specialists and a professional photographer. While educational experts were engaged to review the education structure, content and resources, the initiative itself was not based on a clearly defined theoretical framework. Six sessions are delivered to the client and carer where appropriate at usual wound treatment visits, ideally one session per week. The client and nurse view a multimedia presentation, of approximately 10 min in duration, on the nurse’s tablet computer and then review a summary sheet and complete an activity to reinforce the learning. The client is provided a written booklet of the complete program to keep. The program sessions review what venous disease is and why ulcers occur, venous leg ulcer treatment with compression bandaging, activity and exercise, skin care, nutrition and compression stockings for recurrence prevention (see Table 1).

**Table 1:** Self-management recommendations for Leg Ulcer Prevention Program (LUPP) sessions.

Session	Topic	Self-Management Recommendations
Introduction	Overview of program Compression for healing and recurrence prevention	Ownership of wound and self-management plan
Leg Ulcer Treatment	Compression therapy most clinically effective treatment Promotion of four layer bandaging	Commence compression therapy for treatment Plan for compression following healing
Activity and Exercise	Walking, leg exercises and elevation Use of activity diary	Be active Regular walking (30 min/day) Heel raises and squats (5 sets × 5 repetitions × 3 times/day) Occasional leg elevation (30 min × 3 times/day)
Skin Care	Cleansing Moisturizing Inspection	Daily use of pH neutral cleanser Daily use of pH neutral moisturizer Regular inspection Early reporting of skin integrity concerns
Nutrition and Hydration	Appropriate diet Adequate hydration Tips for healthy eating Nutrition when wounded	Intake guided by Australian Guidelines Minimum of 1 Litre “good” fluid Ensure adequate protein, consider supplementation
Compression stockings for recurrence prevention	Promotion of compression stockings Use of applicators Application and removal technique Stocking care	Wear compression stockings every day Use applicators to assist application and removal Replace stocking every 3 months

Behavior change is influenced by a number of elements such as socioeconomic factors, client and nurse knowledge, access to services, and consistent messages from all members of the healthcare team regarding best practice care. LUPP was not designed to influence all elements. LUPP targeted gains in the areas of knowledge transfer, understanding venous disease and recommended management strategies, and opportunities and activities to practice recommended care.

To deliver LUPP to clients, 60 nurses were trained about the program. The nurse training constituted a 90 min interactive session led by an advanced practicing wound management consultant which was supported by a hard copy training manual. Clients were eligible for the study if they had a medically diagnosed venous leg ulcer and were ineligible only if they did not speak English.

### 2.3. Data Collection

Data collection was attended at five time points. The first was prior to commencing the first LUPP education session and the second was immediately after completing the last LUPP education session; six weeks on average. A subset of study participants (those who healed) were eligible to participate in an RCT comparing the clinical effectiveness of a moderate and a high compression stocking treatment on venous leg ulcer recurrence [48]. These participants would be monitored at a further three time points: upon entry to the RCT, and 13 and 26 weeks from baseline.

In the absence of an existing validated measure, questions relating to health behaviors were custom designed. Items were primarily dichotomous to discern whether each of the recommended behaviors targeted in the program were achieved or not. This study focused on behavior change in the conduct of heel raises and squats, the use of a moisturizer, the use of a soap substitute, increasing the amount of physical activity, and elevating legs. Although the LUPP pilot study considered a number of other domains of health behavior such as nutrition and hydration, as these did not show significant change immediately following the LUPP education these were not assessed for their sustainability. The use of higher levels of compression therapy showed significant increases after the LUPP education, however, this too was not a focus in this sustainability analysis, given the bias introduced because participants were subsequently enrolled in an RCT where compression levels were controlled. The LUPP program and data collection tools were reviewed by some clients and a number of content and education experts prior to commencing the study.

### 2.4. Data Analysis 

IBM^©^ SPSS^©^ Statistics Version 19.0.0 [49] was used to analyze data. Cochran Q tests were conducted to assess if the improvements noted in the post LUPP evaluation data were maintained across the three time points in the RCT.

## 3. Results and Discussion

### 3.1. Sample

Forty-nine people participated in both the LUPP pilot study and the compression stocking RCT. An average timeframe from the beginning of the pilot study (pre LUPP data) through to the 26 week follow-up in the RCT was 252.26 days (*SD *= 49.90; range = 187 to 391). Thus, the sustainability of behavior changes considered in this analysis spans approximately 34 weeks or 8 to 9 months. The average time between completing the LUPP education and recruitment to the RCT was 33.68 days (*SD* = 41.36; range = −1 to 140). The range of days eventuated from the varied healing times of study participants. A description of the sample demographics and wound characteristics is provided. (Table 2 and Table 3).

**Table 2:** Sample description for study participants (*n *= 49).

Demographic/Health Characteristics	Total Sample (*n* = 49)
Gender (% female)	75.5
Age (Years; Ave. ± SD)	76.10 ± 11.91
Nutritional Risk (Ave. ± SD)	12.78 ± 2.02
AACCI (Ave. ± SD) ^	4.78 ± 2.62
Diabetes Mellitus (% yes)	14.3

^ Age Adjusted Charlson Comorbidity Index [50].

**Table 3:** Characteristics of study wound for study participants (*n* = 49).

Wound Characteristics	Total Sample (*n *= 49)
Duration in weeks (Ave. ± SD) *	26.63 ± 20.66
Size (cm^2^; Ave. ± SD) ^	8.32 ± 12.28
Infected during episode (% yes)	59.2

* missing data *n *= 1; extreme outlier excluded *n *= 1; ^ missing data *n *= 1.

### 3.2. Design Sustainability of Health Behavior Changes Overtime of Participants Who Did LUPP

**Figure 1:**
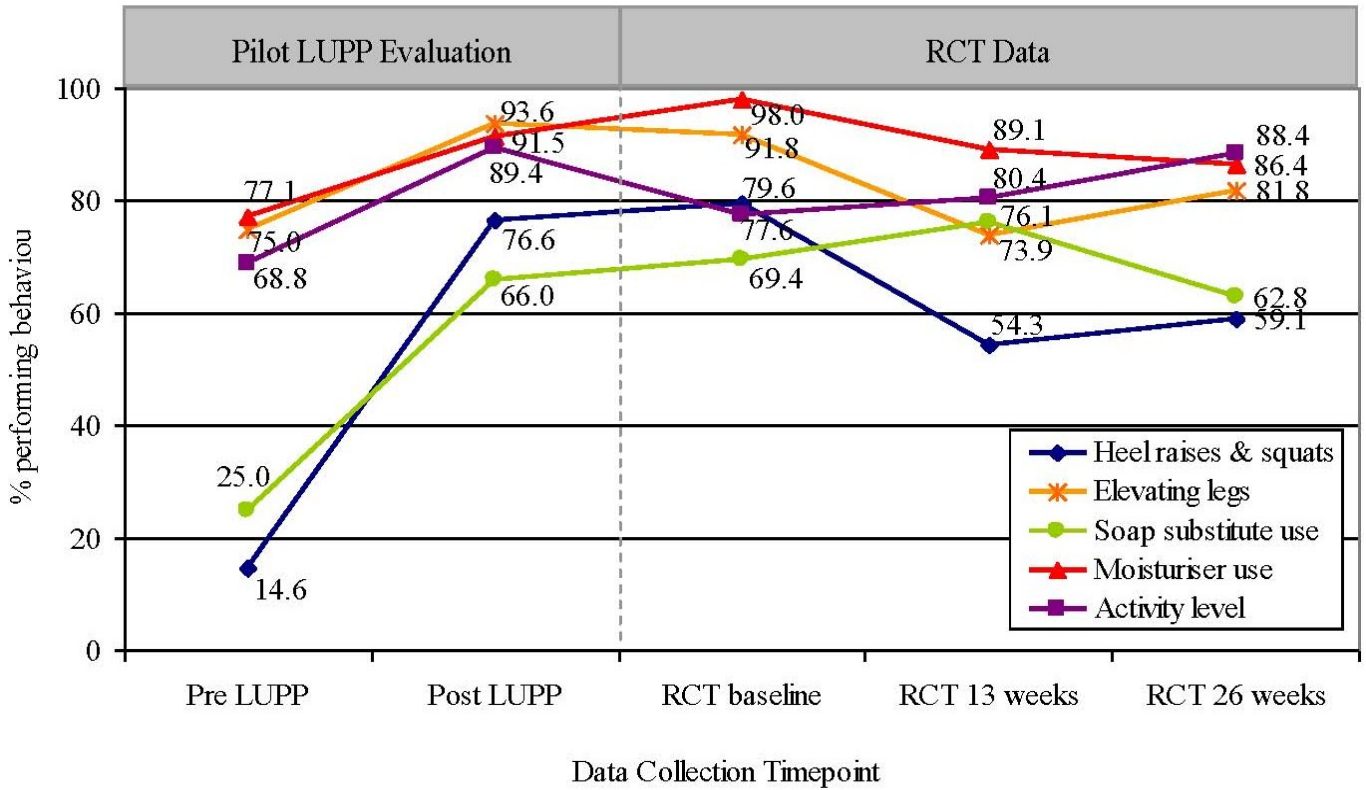
Client’s performance of health behaviors over time.

As shown in Figure 1, while the percentage of participants performing the five health behaviors varied over time, performance was higher for each behavior at the 26 week data collection than before receiving the LUPP education, with behaviors relating to heel raises and squats and use of a soap substitute most different.

There were no significant differences over the time points subsequent to the post LUPP evaluation for activity levels (Cochran Q(3) = 1.364, *p* = 0.714) and the use of a soap substitute (Cochran Q(3) = 1.675, *p* = 0.642). These findings suggest that the improvements that were associated with the LUPP education in these two areas were maintained for at least a 26 week period.

A significant decline in the performance of leg elevation (Cochran Q(3) = 12.607, *p* = 0.005) and heel raises and squats (Cochran Q(3) = 20.500, *p* = 0.000) was observed. The number of study participants elevating their legs declined at the 13 week follow-up to be less than the original pre-LUPP levels before showing a minor increase at the 26 week follow-up. With respect to the conduct of heel raises and squats, an activity that was infrequently performed prior to LUPP but which increased to be employed by more than three quarters of the sample post LUPP, fewer participants were conducting these exercises at the 13 and 26 week RCT follow-up, however uptake remained in excess of half the sample.

Differences in the use of a moisturizer over these time periods approached statistical significance (Cochran Q(3) = 7.696, *p* = 0.053). The conduct of this behavior progressively declined at the subsequent data collections, although it remained one of the most frequently performed health promoting behavior for people with a venous leg ulcer at the 26 week follow-up (86.4%).

## 4. Discussion

This study presents data regarding the sustainability of behavior changes associated with a client e-Learning venous leg ulcer program. The average time between commencing the LUPP education in the pilot study and the final data collection in the RCT was 34 weeks or 8–9 months. The analysis focused on five health behaviors that revealed significant improvements during the original pilot study LUPP evaluation; level of activity, doing heel raises and squats, elevating legs, use of a soap substitute and use of a moisturizer. At the final follow up in the RCT the percentage of participants conducting the behavior in every instance exceeded the pre LUPP levels. However, marked differences in the ongoing conduct of activities were observed.

The only domain in which ongoing improvement was observed was the conduct of physical activity which increased overtime. Moisturizer use, which was already well enacted at baseline, was found to decline over the monitoring period post healing. The use of a soap substitute increased initially post healing only to decline at the last follow up. The number of participants conducting heel raises and squats and elevating their legs initially declined post healing before increasing at the final data collection. Factors shaping behavior maintenance, as discussed earlier, are many and range from the salience of the health issue, integration of the health recommendations with other health priorities [28], and whether the behavior manifests as complimentary or compensatory within the scheme of multiple morbidity management [30].

Ultimately, it is reasonable that the conduct of a health behavior will flux. This is common for most people at other life points so why, in such a complex period of management, should anything other than fluidity be expected. Maintenance of the behaviors that support good health is a juggling act; habits formed might be habits lost repeatedly as a result of health or life events. If the psychological processes of behavior change and behavior maintenance differ [46], then the individual experiencing a health behavior disruption, will need to revisit the behavior change process, re-establish a habit, and then shift into behavior maintenance. 

As Morris and colleagues suggested, skills that can support clients to understand their multiple morbidities and the impact of lifestyle choices can create amalgamations across health recommendations that can foster effective self-management practices [28]. A systematic review regarding ongoing health prompts found that, whilst there was considerable heterogeneity in the “prompts” considered—emails, phone calls, conversations with counsellors, and access to online materials—positive outcomes were more typically observed with the benefits optimized by the prompt frequency and by access to counsellors [51]. Some medical professionals have reported that their medical training has not equipped them to effectively support behavior change [52]. Therefore, given the prevalence of multiple morbidities in the developed world, especially amongst older people, is there sufficient impetus to support routine, ongoing and accessible involvement of clinical specialists and routine clinical appointments to enable people to be informed and prepared to self-manage their mental and physical health?

It is proposed that the variation in health behavior sustainability in this study may relate to an individual’s knowledge and beliefs about the need to persist with health behaviors after healing. In the case of moisturizer and soap substitute use, it may be that the decline in use at 26 weeks reflected that participants felt their skin was in good condition and did not require ongoing use of these products. Alternatively, the ongoing need to conduct calf muscle exercises and to also elevate one’s legs may be a message not well understood, especially if the distinction between a healed leg ulcer and venous insufficiency, which remains an underlying issue after a wound has healed, has not been effectively communicated. It is possible that discussing the activities as part of the RCT data collection process led to renewed awareness and resulting conduct, however, this did not occur at all data collection time points or for all activities suggesting other factors had an impact on the trend of these findings. Nonetheless, emphasis on the importance and need for maintaining health behaviors at the time of healing would help to avoid confusion.

Increased functionality post healing may have also shaped the capacity participants had to perform certain behaviors. For instance, increases in physical activity may reflect improved mobility and conditioning experienced by an individual after their leg ulcer healed. Another important consideration is financial and access barriers that may have affected the individual’s ability to engage in some self-management activities. For example, samples of skin care products were provided to participants as a part of the LUPP education. However, ongoing access to these products maybe limited either for their affordability or availability and may be another factor contributing to the sustainability of these recommended behaviors.

These findings demonstrate that how people implement health behaviors, which are lifelong strategies to improve health and wellbeing, is complex. There has been limited exploration of factors that support the uptake and longevity of behavior changes in relation to wound management and future research should target this area. It is an area of chronic disease management that is also in need of increased evidence and understanding.

The LUPP education focused primarily on knowledge transfer, understanding venous disease and its management, and provided opportunities to engage in self-management practices. These activities included trialing compression stockings, trialing the use of devices that could assist with applying and removing compression stockings, using soap substitutes, and completing an activity diary. These elements, though important, represent part of several elements that generate and sustain behavior change. Newsom, Lions, and Crawford emphasized consideration of how the broader ecology—individual, family, community, country, and world—when attempting to achieve behavior change; the scope of which is a daunting but critical step for the healthcare sector to manage chronic, complex conditions [53]. For effective management of chronic disease to be realized, fundamental shifts in approaches to health are unavoidable. Connection to community, sense of place, and walkability of neighborhoods have been identified by older adults as motivators to physical activity while barriers included health, environment, family and attitudes to physical activity [54]. One quarter of patients with diabetes or heart failure reported family-related factors to be a barrier to self care, an experience more common amongst females [55]. Family barriers were further associated with lower self-efficacy and self-management adherence.

There are a number of limitations to this research study. First, the investigation was made possible by the opportunity presented by two related studies but was not the primary purpose of the study. As such, there are design features missing that would have been incorporated if this was the primary purpose, including more standardized data collection timeframes in between the completion of the LUPP education and data collection attend subsequently. The findings have limited external validity for people with a recently healed venous leg ulcer beyond the characteristics required for entry to the RCT. The pre and post LUPP tools were designed by the Research Team and though they permitted evaluation of the specific aspects of program, they relied on self-report and were not validated instruments. Subsequent evaluations of the LUPP education due to be reported in 2015 have incorporated the use of validated physical activity and nutrition scales and have expanded the response scale for other items beyond a dichotomous classification. The effect of asking for information about health behaviors as part of data collections may have prompted behavior modification potentially altering the “true” gap between the intervention and control groups. Finally, the timeframe for follow up, whilst still adding to existing knowledge in the area of wound management, was still ultimately short.

## 5. Conclusions

Venous leg ulcers are difficult to heal and their high rate of recurrence is a significant worry for client and healthcare provider alike. The LUPP education was established to better inform and support people with venous insufficiency to self-manage their chronic condition. This study observed that behavior changes achieved during a LUPP pilot evaluation did maintain changes in excess of their original level several months after the intervention was complete. However, varied maintenance patterns emerged for these behaviors. Approaches to multiple component self-management training and maintenance support is an area where further research is in need not only in relation to wound management but chronic disease more generally.
